# An ensemble of bias-adjusted CMIP6 climate simulations based on a high-resolution North American reanalysis

**DOI:** 10.1038/s41597-023-02855-z

**Published:** 2024-01-11

**Authors:** Juliette Lavoie, Pascal Bourgault, Trevor James Smith, Travis Logan, Martin Leduc, Louis-Philippe Caron, Sarah Gammon, Marco Braun

**Affiliations:** Ouranos, Montreal, H3A 1B9 Canada

**Keywords:** Climate-change impacts, Projection and prediction, Climate change

## Abstract

ESPO-G6-R2 v1.0 is a set of statistically downscaled and bias-adjusted climate simulations based on the Coupled Model Intercomparison Project 6 (CMIP6) models. The dataset is composed of daily timeseries of three variables: daily maximum temperature, daily minimum temperature and daily precipitation. Data are available from 1950 to 2100 over North America. The simulation ensemble is comprised of 14 models driven by two emissions scenarios (SSP2-4.5 and SSP3-7.0). In this paper, we describe the workflow used for the bias-adjustment, which relies on the detrended quantile mapping method and the Regional Deterministic Reforecast System (RDRS) v2.1 reference dataset. Using the framework defined in the VALUE project, we show the improvements made by the bias-adjustment on marginal, temporal and multivariate aspects of the data. We also verify that the bias-adjusted climate data have similar climate change signal to the original climate model simulations. Finally, we provide guidance to users on how to use this dataset.

## Background & Summary

The need to adapt to climate change is present in a growing number of sectors, leading to an increase in the demand for authoritative, easily accessible, quality controlled climate information. In order to meet this growing demand and to support numerous Vulnerability, Impact, and Adaptation (VIA) studies, Ouranos, a Quebec-based climate change adaptation research consortium, produced a set of operational multipurpose bias-adjusted climate simulations referred to as *Ensemble de Simulations Post-traitées d’Ouranos* (ESPO). This paper presents the ESPO-G6-R2 v1.0 dataset. The “-G6” suffix refers to the sixth version of the Global (G) Coupled Model Intercomparison Project (CMIP)^1^ models, used as inputs, while the “-R2” suffix refers to the Regional Deterministic Reforecast System (RDRS) v2.1 reanalysis^2^, used as an observational reference for the bias-adjustment. The ESPO-G6-R2 dataset serves multiple purposes: it is needed internally for climate change adaptation projects, supports the organization’s climate portal *Portraits Climatiques* (https://portraits.ouranos.ca), and is additionally made available to collaborators and external users through the PAVICS (https://pavics.ouranos.ca) platform (a hosted JupyterLab analysis environment with an associated THREDDS data server). This dataset is an updated version of a previous dataset based on CMIP5 simulations^3^.

**Fig. 1:**
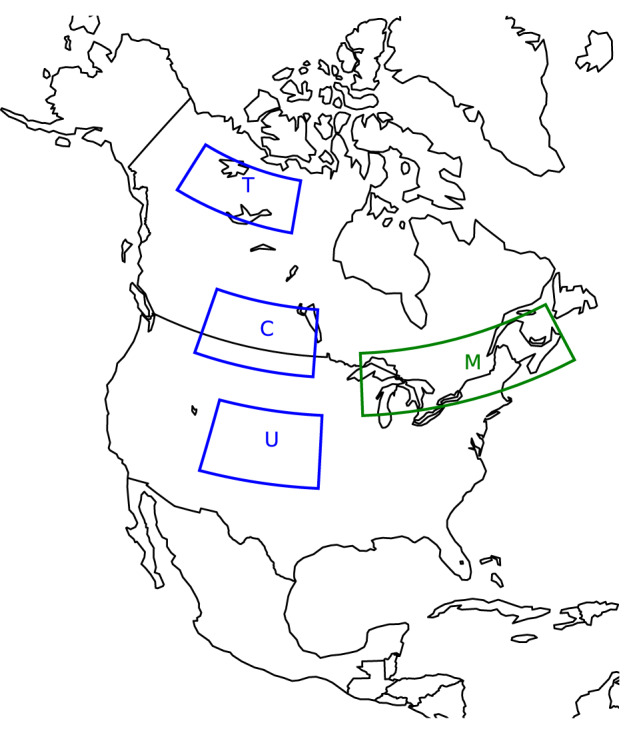
The full North American domain of the dataset. The Magtogoek region used for validation is shown with the green contour. The Tlicho, Cree and Ute regions are shown with blue contours and the first letter of the region name. These three regions have the same number of grid cells.

To build the ESPO-G6-R2 v1.0 dataset, the original CMIP6 simulations were statistically downscaled and bias-adjusted using a variant of the detrended quantile mapping method and the RDRS v2.1 dataset. This reanalysis product was created by Environment and Climate Change Canada (ECCC) using the Regional Deterministic Reforecast System (RDRS) to downscale the Global Deterministic Reforecast System (GDRS) initialized with ERA-Interim^4^. The system is also coupled with the Canadian Land Data Assimilation System (CaLDAS)^5–7^ and Canadian Precipitation Analysis (CaPA)^8–11^. RDRS v2.1 and, consequently, the ESPO-G6-R2 dataset, covers North America (Fig. 1) at a resolution of 0.09° on a rotated uniform latitude–longitude grid. While RDRS v2.1 provides data over both land and ocean, ESPO-G6-R2 adopts the same domain but was only produced over land with a narrow buffer along the coasts. Ocean grid points were masked prior to bias-adjustment. The dataset includes daily minimum temperature (tasmin), daily maximum temperature (tasmax) and daily precipitation (pr) for the 1950–2100 period for the emissions scenarios SSP2-4.5 and SSP3-7.0^12^. Only original simulations produced with models having a Transient Climate Response (TCR) within the Intergovernmental Panel on Climate Change (IPCC)-defined likely range (1.4–2.2 °C^13^) were used to create the ensemble (Table 1), as was suggested by Hausfather *et al*.^14^. A single member per model is used to create the ensemble.

**Table 1:** Climate simulations included in ESPO-G6-R2 v1.0.

Institution	Model	Member	Original Resolution (lon × lat in°)
CSIRO-ARCCSS	ACCESS-CM2^37^	r1i1p1f1	1.9 × 1.2
CSIRO	ACCESS-ESM1-5^38^	r1i1p1f1	1.9 × 1.2
BCC	BCC-CSM2-MR^39^	r1i1p1f1	1.1 × 1.1
CNRM-CERFACS	CNRM-ESM2-1^40^	r1i1p1f2	1.4 × 1.4
CMCC	CMCC-ESM2^41^	r1i1p1f1	1.2 × 0.9
CAS	FGOALS-g3^42^	r1i1p1f1	2.0 × 2.0
NOAA-GFDL	GFDL-ESM4^43^	r1i1p1f1	1.2 × 1.0
INM	INM-CM5-0^44^	r1i1p1f1	2.0 × 1.5
NIMS-KMA	KACE-1-0-G^45^	r1i1p1f1	1.9 × 1.2
MIROC	MIROC6^46^	r1i1p1f1	1.4 × 1.4
DKRZ	MPI-ESM1-2-HR^47^	r1i1p1f1	0.9 × 0.9
MPI-M	MPI-ESM1-2-LR^48^	r1i1p1f1	1.9 × 1.9
MRI	MRI-ESM2-0^49^	r1i1p1f1	1.1 × 1.1
NCC	NorESM2-LM^50^	r1i1p1f1	2.5 × 1.9

In this paper, we begin by describing the bias-adjustment method used to create this dataset. Next, we provide information on the data and how to acquire it. We subsequently validate the dataset, in part by following the VALUE project framework^15^. Finally, we provide recommendations for effective usage of the data.

## Methods

### Data extraction

The original CMIP6 simulations^16^ (Table 1) were downloaded from the Earth System Grid Federation (ESGF) archive using the *synda* Python library (now replaced by *esgpull*; https://esgf.github.io/esgf-download) and the North American domain was extracted. The reference dataset (RDRS v2.1 reanalysis^2^) was downloaded from CaSPAr (https://caspar-data.ca)^17^ under ECCC Data Servers End-use Licence (https://eccc-msc.github.io/open-data/licence/readme_en/). As we are not interested in data over the ocean, we selected all grid cells that have a sea area fraction less than one and we added a buffer of one grid cell along coastlines.

### Regridding

In order to perform the bias-adjustment, all simulation land and ocean cells were interpolated onto the masked RDRS v2.1 grid using the bilinear method. Because of the large difference in resolution between the simulations and RDRS v2.1, the regridding is done in cascade, from the original grid to a 1° regular grid, to a 0.5° regular grid, to the final RDRS v2.1 rotated 0.09° resolution grid.

### Bias-adjustment

The ESPO-G6-R2 v1.0 bias-adjustment procedure uses a variant of the detrended quantile mapping method, as provided by *xclim* (https://xclim.readthedocs.io)^18^ and described by many authors^19,20^. The procedure is univariate (applied to each variable individually), acts independently on the trends and the anomalies, and is applied iteratively on each day of the year as well as at each grid point.

#### Variables

Adjustments are applied separately for each of the three variables. Because adjusting tasmax and tasmin independently can lead to physical inconsistencies in the final data (i.e., cases with tasmin > tasmax^21,22^), we instead applied the bias correction to the daily temperature range (or amplitude; dtr = tasmax - tasmin) in addition to tasmax and pr. tasmin was reconstructed after the bias-adjustment.

While tasmax has no physical bounds in practice, this is not the case for pr and dtr which are bounded by zero. To apply this constraint in practice, the adjustment process is additive for tasmax and multiplicative for pr and dtr^23^. The multiplicative approach prevents values to drop below zero.

#### Grouping and calendar

The bias-adjustment is applied to each day of the year and each grid point independently. To render the procedure more robust, a window of 31 days centred on the current day of the year was used for the calibration (training step). For example, the adjustment for February 1 was calibrated using data from January 15 to February 15, over the 30 years of the reference period. In order to avoid having four (4) times fewer data points for the 366th day of the year during leap years, we converted all inputs to a “noleap” calendar by removing data on the 29th of February. For simulations using the “360_day” calendar, the simulations were untouched, but the RDRS v2.1 data was converted to that calendar by removing days at regular intervals.

#### Detrending

We first computed the averages (−) and anomalies (′) of the RDRS v2.1 reference data (*Y*_*r*_) and regridded simulations over the 1989–2018 reference period (*X*_*r*_), the most recent 30-year period in the RDRS v2.1 dataset, for each day of the year and each grid point. The subscripts indicate the period (in this case, reference). As mentioned above, anomalies are computed either additively or multiplicatively, depending on the variable:

1 $${Y}_{r}=\{\begin{array}{cc}{\bar{Y}}_{r}+{Y}_{r}^{{\rm{{\prime} }}} & {\rm{f}}{\rm{o}}{\rm{r}}\,{\rm{t}}{\rm{a}}{\rm{s}}{\rm{m}}{\rm{a}}{\rm{x}}\\ {\bar{Y}}_{r}\cdot {Y}_{r}^{{\rm{{\prime} }}} & {\rm{f}}{\rm{o}}{\rm{r}}\,{\rm{d}}{\rm{t}}{\rm{r}},{\rm{p}}{\rm{r}}\end{array}$$

and similarly for *X*, $\bar{X}$ and *X*′.

Instead of a simple moving mean, the simulation was detrended with a locally weighted regression (LOESS^24^) over the full 1950–2100 simulation period (*X*_*s*_). We chose this method for the slightly heavier weights given to the centre of the moving window, thus reducing the impacts of abrupt inter-annual changes on the trend and anomalies. It also performs better near the edge of the timeseries. The LOESS window had a 30-year width and a tricube shape, while the local regression was of degree 0 and only one robustness iteration was performed. The LOESS detrending was applied on each day of the year after averaging over a 31-day window, yielding the trend ${\bar{X}}_{s}$ and the residuals ${X}_{s}^{{\prime} }$. Here again, the process can be additive or multiplicative.

#### Adjustment of the residuals

With ${F}_{{Y}_{r}^{{\prime} }}$ and ${F}_{{X}_{r}^{{\prime} }}$ the empirical cumulative distribution functions (CDF) of ${Y}_{r}^{{\prime} }$ and ${X}_{r}^{{\prime} }$ respectively, an adjustment factor function was first computed:

2 $${A}_{+}(q):={F}_{{Y}_{r}^{{\prime} }}^{-1}\left(q\right)-{F}_{{X}_{r}^{{\prime} }}^{-1}\left(q\right)\quad \quad \quad {A}_{\times }(q):=\frac{{F}_{{Y}_{r}^{{\prime} }}^{-1}\left(q\right)}{{F}_{{X}_{r}^{{\prime} }}^{-1}\left(q\right)}$$

where *q* is a quantile (in range [0, 1]), *A*_+_(*q*) is the additive function used with tasmax and *A*_×_(*q*) the multiplicative function used with pr and dtr. The CDFs were estimated from the thirty (one for each year) 31-day windows. In the implementation, during the training step, maps of *A* were saved to disk by sampling *q* with 50 values, going from 0.01 to 0.99 by steps of 0.02. The adjustment for each day was then as follows:

3 $${X}_{s}^{* {\prime} }={X}_{s}^{{\prime} }+{A}_{+}\left({F}_{{X}_{r}^{{\prime} }}\left({X}_{s}^{{\prime} }\right)\right)\quad \quad \quad {X}_{s}^{* {\prime} }={X}_{s}^{{\prime} }\cdot {A}_{\times }\left({F}_{{X}_{r}^{{\prime} }}\left({X}_{s}^{{\prime} }\right)\right),$$

where ${X}_{s}^{* }$ is the bias-adjusted simulation over the simulation period (1950–2100). Nearest neighbour interpolation was used to map ${F}_{{X}_{r}^{{\prime} }}({X}_{s}^{{\prime} })$ to the 50 values of *q*. Constant extrapolation was used for values of ${X}_{s}^{{\prime} }$ outside the range of ${X}_{r}^{{\prime} }$.

#### Adjustment of the trend

In the training step, a simple scaling or offset factor was computed from the averages:

4 $${C}_{+}={\bar{Y}}_{r}-{\bar{X}}_{r}\quad \quad \quad {C}_{\times }=\frac{{\bar{Y}}_{r}}{{\bar{X}}_{r}}$$

This factor was then applied to the trend in the adjustment step:

5 $${\bar{X}}_{s}^{* }={\bar{X}}_{s}^{* }+{C}_{+}\quad \quad \quad {\bar{X}}_{s}^{* }={\bar{X}}_{s}\cdot {C}_{\times }$$

Finally, the bias-adjusted timeseries ${X}_{s}^{* }$ for a given day of the year, grid point, and variable is simply the sum or product of these two terms:

6 $${X}_{s}^{* }={\bar{X}}_{s}^{* }+{X}_{s}^{* {\prime} }\quad \quad \quad {X}_{s}^{* }={\bar{X}}_{s}^{* }\cdot {X}_{s}^{* {\prime} }$$

#### Pre-processing for multiplicative bias-adjustment

Two extra steps are added in the multiplicative adjustment procedure. First, it should be noted that the multiplicative mode is prone to division by zero, especially with precipitation where values of zero are quite common. This problem was resolved by modifying the inputs of the calibration step, where the zeros of precipitation were replaced by random values between zero (excluded) and 0.01 *mm*/*d*. Even though in dtr values close to 0 °C are rare, the dtr timeseries was also modified for values under 0.0001 °C with random values above 0 °C.

Second, Themeßl *et al*.^25^ observed that models having higher dry-day frequency than the reference (here, RDRS v2.1) can produce additive adjustment factors that map dry days to wet days, resulting in a wet bias. With a multiplicative adjustment and the injection of very small random values in the first pre-processing step, this problem transforms into the generation of large adjustment factors for those extra dry days where the very small (dry) simulation quantile divides realistic (wet) RDRS v2.1 quantile (see *A*_×_ in Eq. 2). These aberrant factors generate unphysical values in the adjustment procedure. To remove this bias, a second pre-processing step was applied. The frequency adaptation method, as proposed by Themeßl *et al*.^25^, finds the fraction of “extra” dry days:

7 $$\Delta {P}_{dry}=\frac{{F}_{{X}_{r}}(D)-{F}_{{Y}_{r}}(D)}{{F}_{{X}_{r}}(D)}$$

where *D* is the dry-day threshold, taken here to be 1 *mm*/*d*. During the training step, a fraction Δ*P*_*dry*_ of dry days was transformed into wet days by injecting random values taken in the interval $\left[D,{F}_{{Y}_{r}}^{-1}\left({F}_{{X}_{r}}(D)\right)\right]$ (the precipitation value in RDRS v2.1 at the first quantile with precipitation in the simulation). Hence, adjustment factors are calculated separately for dry days and wet days. Note that, in the inverse case, where RDRS v2.1 would have a higher dry-day frequency than the models, the small number is in the numerator and only a few wet days are mapped to dry days. This is not a problem.

Both pre-processing functions were applied only on the calibration step inputs (*Y*_*r*_ and *X*_*r*_) before the division between average and anomalies (Eq. 1). As such, only the adjustment factors were impacted while there were no explicitly injected precipitation values in the final bias-adjusted simulations.

## Data Records

The reference for the ESPO-G6-R2 dataset is 10.5281/zenodo.7877330^26^. The dataset is available through a Thematic Real-time Environmental Distributed Data Services (THREDDS) at the following link: https://pavics.ouranos.ca/twitcher/ows/proxy/thredds/catalog/birdhouse/ouranos/ESPO-G/ESPO-G6-R2v1.0.0/catalog.html.

The dataset is stored in NetCDF files. Each netCDF file (1 GB) contains 4 years of data for one model, one variable and one emissions scenario. Loaded together they create the full dataset containing timeseries from 1950 to 2100 for daily maximum temperature, daily minimum temperature and daily precipitation over North America at a 0.09° resolution on a rotated uniform latitude-longitude grid. Information about the grid is included in the attributes of the rotated_pole coordinate of each file.

The ensemble analysed in this paper includes the climate models listed in Table 1 for emissions scenarios SSP2-4.5 and SSP3-7.0. We also provide data for additional models, including models which have a TCR outside the likely range defined in the latest IPCC report, as well as simulations following the emissions scenarios prescribed by SSP 5–8.5. All CMIP6 models are made available under the CC-BY 4.0 License.

## Technical Validation

Here, we present a succinct evaluation of ESPO-G6-R2 v1.0.

### Health checks

First, health checks were conducted to verify that the datasets were free of unphysical values. The five checks are:

1. No negative pr
2. No tasmin above tasmax
3. No tasmax above 60 °C
4. No tasmin below −70 °C
5. No pr above 1650 mm/d

All the bias-adjusted simulations in the ensemble passed the first three checks. The fourth check on minimum temperature is based on the minimum recorded temperature in the northern hemisphere (−69.6 °C in Klinck, Greenland^27^) and revealed some issues. As CMIP6 models have very large grid cells, regions that are considered land on the RDRS v2.1 grid might correspond to an ocean grid cell in the model. In regions with sea ice, this can lead to issues with dtr being very small over water and larger over ice. Because the quantile mapping methodology is not designed to address these non-linear effects, this can lead to very large dtr and translate into non-physical tasmin values (very close to 0 K). This problem was detected near the coasts of Alaska and Greenland for the BCC-CSM2-MR and GFDL-ESM4 models. Instances of tasmin smaller than 100 K (−173.15 °C) have been replaced by NaN values.

There are still a few extremely rare cases (6 × 10^−6^% of all data points) where the minimum temperature is lower than the observed threshold, although we note that this threshold is also exceeded in the original simulations. We do not replace these extremes by NaN values. The last check is based on the highest recorded 1-day precipitation in the Northern Hemisphere (1633.98 mm in Isla Mujeres, Mexico)^28,29^. This threshold is only exceeded in 1 × 10^−7^% of the dataset.

### Inspection of the projected change

The goal of our bias-adjustment, which implicitly includes statistical downscaling, is to combine the climate change signal from the original simulation with the statistical information from the present climate at the fine spatial scale available in the reference dataset, RDRS v2.1. In this section, we validate that the bias adjustment did not impact the multimodel median projected changes in mean temperature and mean precipitation, whereas in the next section, we compare the bias-adjusted simulations with RDRS v2.1 for the present climate.

**Fig. 2:**
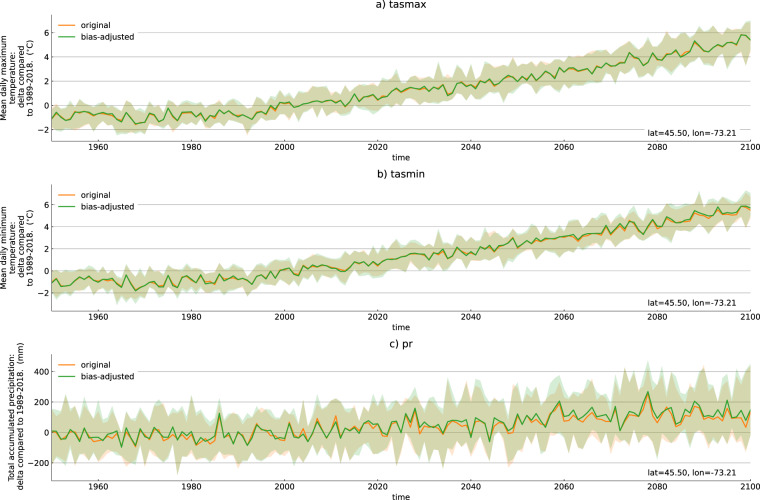
Original and bias-adjusted ensemble median and spread of the change in three annual indices: mean daily maximum temperature, mean daily minimum temperature, and total precipitation. The change is computed with reference to the 1989–2018 period mean. The time series are computed for one grid cell near Montreal, Canada, for SSP3-7.0. The solid line represents the 50th percentile of the ensemble and the shading represents the range from 10th to 90th percentile.

We start by inspecting the annual time series of the three variables, expressed as the difference with respect to the 1989–2018 climatology, for a grid cell located near Montreal, Canada.We show results for SSP3.7-0 only, but they are similar for SSP2-4.5. Figure 2 shows that the median change of the original regridded simulations (*X*_*r*_) and the median change of the regridded and bias-adjusted simulations (${X}_{r}^{* }$) are similar, confirming that the climate change signal is not impacted by the bias-adjustment. From this figure, we can also see that the ensemble spread is not impacted by the bias adjustment, as that spread (90th percentile - 10th percentile) is similar in both ensembles. F-tests confirm that the variance of the original and bias-adjusted distributions are not statistically different.

**Fig. 3:**
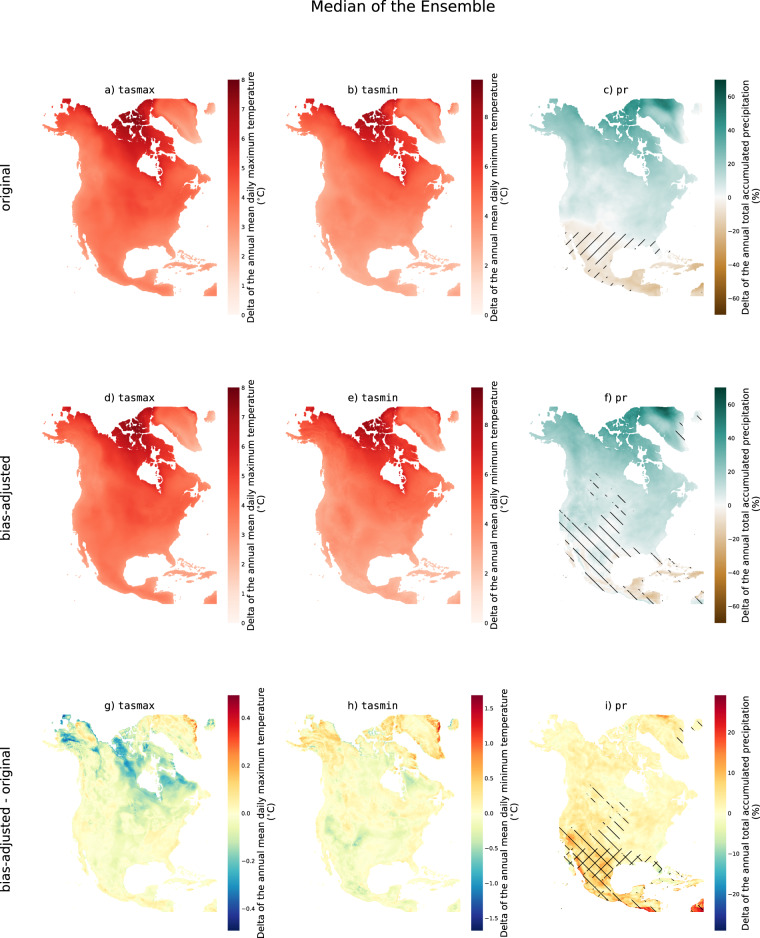
Ensemble median of the difference between the 2071–2100 climatology and the 1989–2018 climatology of tasmax (first column), tasmin (second column), and pr (third column) for SSP3-7.0. The first row shows the results for the original simulations, the second row shows the results for the bias-adjusted simulations and the third row shows the difference between the two. Hatched regions do not have model agreement on the sign of the projected change.

**Fig. 4:**
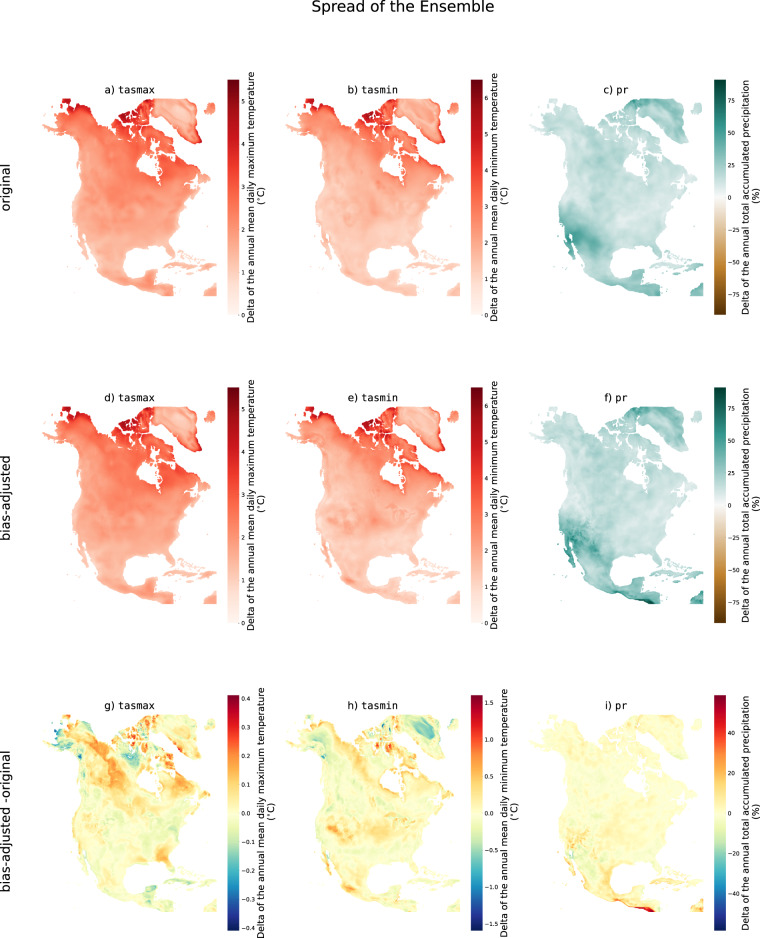
Ensemble spread (difference between the 90th and 10th percentile) of the difference between the 2071–2100 climatology and the 1989–2018 climatology of tasmax (first column), tasmin (second column), and pr (third column) for SSP3-7.0. The first row shows the results for the original simulations, the second row shows the results for the bias-adjusted simulations and the third row shows the difference between the two.

We now extend this analysis to the entire domain. Figure 3 shows the difference between the 2071–2100 and 1989–2018 climatological means for both the original and the bias-adjusted ensemble. For the bias-adjusted simulations (Fig. 3d,e), the average warming is 4.1 °C for tasmax and 4.3 °C for tasmin, with the northern region warming more than the southern region. The spread of the ensemble is also larger in the north (Fig. 4). These results are consistent with the signal obtained in the original simulations (Figs. 3a,b, 4a,b).

For precipitation, the bias-adjusted simulations project an increase in precipitation over most of the domain, except for some small regions across the Caribbean islands and southern Mexico, where models don’t agree on the sign of the change (Fig. 3). Here, Approach B of the IPCC report is used to define model agreement (Cross-Chapter Box Atlas.1), wherein agreement is found if at least 80% of the models have the same sign for a given projected change^30^. This is similar to the original simulations, except that the region without model agreement expands slightly. As noted previously, the spread of the ensemble is similar in the original and bias-adjusted simulations (Fig. 4c,f).

In general, the climate change signal is well-preserved in the bias-adjusted simulations. Nevertheless, since this dataset was generated using GCMs with spatial resolutions coarser or equal to 100 km, it is still essential to exercise caution when interpreting climate change patterns at finer scales.

### Evaluation Through VALUE Diagnostics

In this section, we evaluate the capacity of our bias-adjustment method to improve the representation of the RDRS v2.1 reference climate with respect to the original simulations using the diagnostic framework developed in the VALUE project^15^. Each diagnostic is based on a property (called “indices” in the VALUE project) and a measure. Properties evaluate a dataset’s statistical characteristics by collapsing the time axis and are divided into three aspects: marginal (related to the distribution), temporal (related to the annual cycle, spells and transitions) and multivariate (related to the relation between variables). Measures evaluate the differences in a given property between two datasets.

We compute properties for RDRS v2.1 (*Y*_*d*_), the original regridded simulations (*X*_*d*_), and the regridded and bias-adjusted simulations (${X}_{d}^{* }$) over the 1981–2010 diagnostic (d) period. Measures are then calculated between the RDRS v2.1 reference and the original simulation, as well as between the RDRS v2.1 reference and the bias-adjusted simulation. The complete list of computed diagnostics is provided in Table 2. More details on the implementation of the properties and measures, including references and the code used to compute them, can be found within the modules *xclim.sdba.properties* and *xclim.sdba.measures*.

**Table 2:** Diagnostics used to assess the performance of ESPO-G6-R2 v1.0, including the full name of the property, the short name used in figures, the variable(s) on which the property is calculated, the measure associated with the property, and the aspect evaluated by the property.

Property	Short name	Variables	Measure	Aspect
Mean	mean	tasmax, tasmin, pr,dtr	bias	marginal
First percentile	q01	tasmax, tasmin	bias	marginal
95th percentile	q95	pr	bias	marginal
99th percentile	q99	tasmax, tasmin, pr, dtr	bias	marginal
Dry spell frequency	dry_spell_freq	pr	bias	marginal
Amplitude of the annual cycle	aca	tasmax, tasmin	bias	temporal
Relative amplitude of the annual cycle	aca	pr	ratio	temporal
Dry-Wet Transition	dry_wet	pr	bias	temporal
Wet-Wet Transition	wet_wet	pr	bias	temporal
Maximum length of dry spell	max_dry_spell	pr	bias	temporal
Maximum length of warm spell	max_warm_spell	tasmax	bias	temporal
Inter-variable correlation (Spearman)	corr	tasmin-tasmax, pr-tasmax	bias	multivariate

The diagnostics are computed using daily time series over the 1981–2010 diagnostic period for each model. Note that the diagnostic period is different from the reference training period (1989–2018) in order to maximize the number of independant years used for validation. The diagnostics shown here are calculated over a subregion (green contour in Fig. 1) in order to reduce the computational load and to focus on the region of interest for the majority of Ouranos’ stakeholders. We will refer to this region as Magtogoek, after the Algonquin word for the St-Lawrence River^31,32^. Additionally, to increase confidence in the dataset for the rest of the domain, we compute diagnostics on three smaller regions with distinct climates and show the results in the Supplementary Information. The regions are labelled Tlicho, Cree and Ute after traditional native territories^33^ and are shown with blue contours on Fig. 1.

**Fig. 5:**
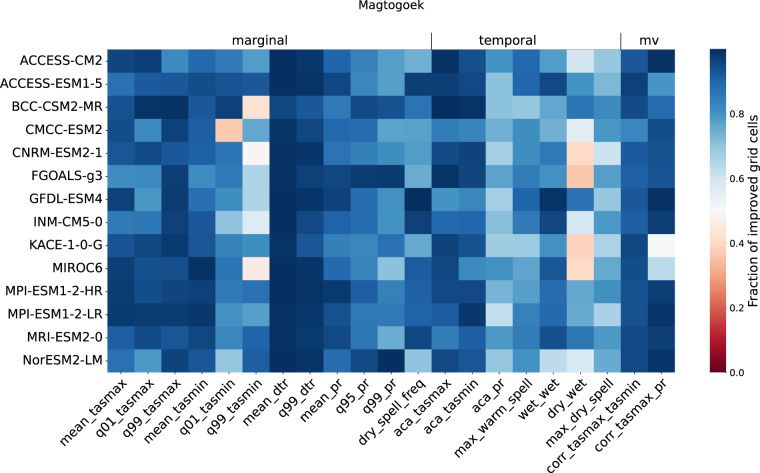
Heatmap of the percentage of improved grid cells (*IMP*) between the original and the bias-adjusted simulations over the Magtogoek region for SSP3-7.0. The columns represent the properties (identified by their short name) while the rows represent the climate models. A value close to 1 means that the bias-adjustment improves the given property over most of the area compared to the original simulation, while a value close to 0 means that the original simulation provides a better performance.

**Fig. 6:**
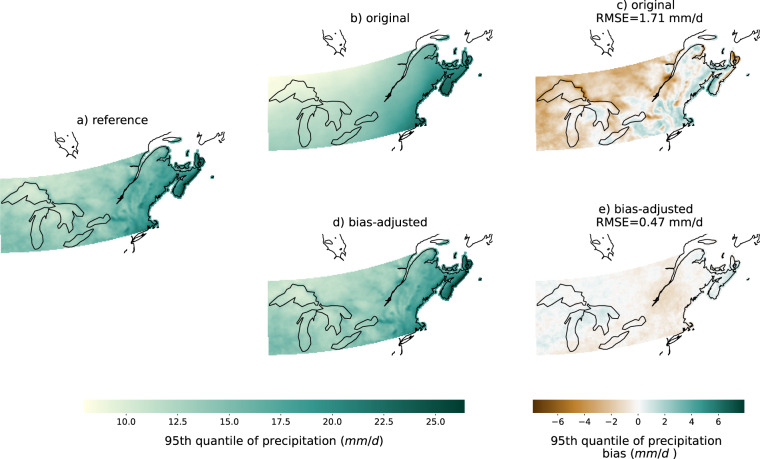
95th quantile of the precipitation (property in a, b, d) and its biases (measure in c, e) during the 1981–2010 period using the RDRS v2.1 reference (**a**), the original KACE-1-0-G SSP3-7.0 simulation (**b,c**) and the bias-adjusted KACE-1-0-G SSP3-7.0 simulation (**d,e**). Note that plot c and d show biases, while the title displays the RMSE.

In order to summarize the analysis across all models, emissions scenarios and properties, Fig. 5 shows the fraction of improved grid cells (*IMP*) over the Magtogoek region. *IMP* is calculated as the fraction of grid cells that results in a better measure in the bias-adjusted simulation (e.g. Figure 6e) compared to the original simulation measure (e.g. Figure 6c), which means either a smaller bias or a ratio closer to 1. *IMP* is defined as

8 $$IMP=\frac{1}{N}\sum _{i,j}{I}_{i,j}\quad \quad {\rm{where}}\quad \quad {I}_{i,j}=\left(\begin{array}{ll}\left(\begin{array}{cc}1 & {\rm{if}}\left|{M}_{i,j}^{sim}\right| > \left|{M}_{i,j}^{scen}\right|\\ 0 & {\rm{if}}\left|{M}_{i,j}^{sim}\right| < \left|{M}_{i,j}^{scen}\right|\end{array}\right. & {\rm{if}}\;{\rm{M}}\;{\rm{is}}\;{\rm{a}}\;{\rm{bias}}\\ \left(\begin{array}{cc}1 & {\rm{if}}\left|{M}_{i,j}^{sim}-1\right| > \left|{M}_{i,j}^{scen}-1\right|\\ 0 & {\rm{if}}\left|{M}_{i,j}^{sim}-{\rm{1}}\right| < \left|{M}_{i,j}^{scen}-{\rm{1}}\right|\end{array}\right. & {\rm{if}}\;{\rm{M}}\;{\rm{is}}\;{\rm{a}}\;{\rm{ratio}}\end{array}\right.$$

where *M* is the measure of the bias between the original or bias-adjusted simulation and the RDRS v2.1 reference and *N* is the number of grid cells (*i*, *j*) in the region. The advantage of this method is that it allows for the comparison of all properties using the same unit of measurement. Figure 5 shows that ESPO-G6-R2 v1.0 provides an improvement over the original simulations for most properties, as evidenced by the majority of values above 0.5. It is important to note that the underlying assumption of this analysis is that RDRS v2.1 used reflects the “ground truth”. As such, any biases that might be present in this dataset are passed along to the bias-adjusted simulations and are undetectable with this analysis. The quality of the RDRS v2.1 itself is beyond the scope of this paper. We encourage users of the dataset to verify the performance of RDRS v2.1 for their region and application.

The following sections further elaborate on the performance of the three aspects evaluated (marginal, temporal, multivariate). We show the maps of three properties for one model as an illustrative examples. The results are not exactly the same for every model, but the general conclusion and explanations remain the same. The maps also demonstrate that the fine-scale spatial features are better represented in the bias-adjusted dataset. We still note that there can be inflation in the dataset as is the case for most quantile mapping methods that rely on a simple spatial downscaling^34^. This means that a day of extreme precipitation in a GCM grid cell will lead to more extreme precipitation in all finer grid cells of the bias-adjusted simulation that were contained in the original grid cell, instead of only in a few grid cells. Supplementary Information shows the impact on one grid cell as an example. We recommend caution using this dataset if spatial features of daily precipitation have a high impact on the user’s application. The use of regional climate models as the input instead of GCMs could help alleviate this problem as the resolution difference would be less important.

Finally, for each property, we compute the the root-mean square error (RMSE) between the RDRS v2.1 reference and original simulation as well as between the RDRS v2.1 reference and the bias-adjusted simulation to help user better assess the error before and after bias-adjustment. Table 3 shows the model average RMSE for each property and regions. The RMSE for the bias-adjusted simulations are nearly always smaller than for the original simulation.

**Table 3:** Ensemble mean RMSEs for each property and each region (Magtogoek, Tlicho, Ute and Cree) for SSP3-7.0 between the RDRS v2.1 reference and the original simulation (OR) and between the RDRS v2.1 reference and the bias-adjustment simulation (BA).

*Regions*	M	M	T	T	U	U	C	C
*Simulations*	OR	BA	OR	BA	OR	BA	OR	BA
mean_tasmax (°C)	1.84	**0.25**	1.87	**0.24**	2.62	**0.20**	1.89	**0.29**
q01_tasmax (°C)	4.06	**0.67**	3.10	**0.59**	2.97	**0.40**	2.67	**0.45**
q99_tasmax (°C)	4.95	**0.43**	3.88	**0.35**	4.36	**0.48**	4.23	**0.63**
mean_tasmin (°C)	2.24	**0.25**	2.69	**0.31**	3.54	**0.17**	2.80	**0.25**
q01_tasmin (°C)	6.05	**1.84**	4.04	**1.34**	6.25	**2.49**	5.71	**2.02**
q99_tasmin (°C)	2.30	**1.00**	2.29	**0.83**	3.51	**1.15**	2.17	**1.01**
mean_dtr (°C)	2.73	**0.09**	1.91	**0.11**	3.71	**0.14**	2.72	**0.12**
q99_dtr (°C)	4.27	**0.33**	3.32	**0.34**	5.25	**0.28**	4.24	**0.29**
mean_pr (mm d^−1^)	0.43	**0.07**	0.31	**0.04**	0.56	**0.10**	0.47	**0.05**
q95_pr (mm d^−1^)	1.97	**0.39**	1.18	**0.21**	2.59	**0.52**	1.86	**0.29**
q99_pr (mm d^−1^)	4.55	**1.16**	2.81	**0.73**	7.04	**1.39**	4.47	**0.96**
dry_spell_freq	0.07	**0.02**	0.09	**0.01**	0.14	**0.01**	0.12	**0.01**
aca_tasmax (°C)	5.49	**0.42**	5.04	**0.80**	5.39	**0.44**	4.09	**0.69**
aca_tasmin (°C)	4.76	**0.54**	4.31	**0.70**	2.78	**0.52**	3.14	**0.68**
aca_pr (%)	19.83	**9.99**	41.44	**13.31**	40.61	**13.61**	51.82	**17.66**
max_warm_spell (days)	15.87	**13.57**	**10.71**	10.85	16.44	**13.57**	13.84	**11.20**
wet_wet	0.07	**0.02**	0.08	**0.01**	0.12	**0.01**	0.09	**0.01**
dry_wet	0.01	**0.01**	0.02	**0.01**	0.02	**0.01**	0.03	**0.01**
max_dry_spell (days)	7.67	**7.23**	18.58	**15.24**	23.22	**17.17**	24.52	**16.16**
corr_tasmax_tasmin	0.03	**0.00**	0.01	**0.00**	0.05	**0.01**	0.03	**0.00**
corr_tasmax_pr	0.16	**0.07**	0.25	**0.05**	0.17	**0.07**	0.14	**0.06**

The smallest RMSE is in bold.

#### Marginal aspect

As expected, the detrended quantile mapping method performs much better than the original simulation for the marginal aspects of tasmax and pr. This is not surprising since this method adjusts each quantile separately. To illustrate, we show in Fig. 6 that the bias in the 95th percentile has been near-completely removed in the bias-adjusted simulation. This matches the corresponding model average *IMP* of 88%. There are also large improvement in RMSE.

**Fig. 7:**
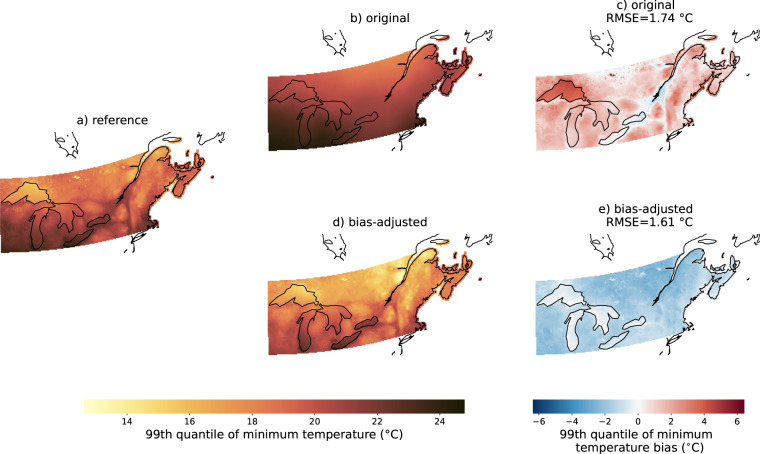
Same as Fig. 6, but for the 99th quantile of the daily minimum temperature and MIROC6 SSP3-7.0.

The results are slightly different for tasmin, which was not adjusted directly. Indeed, in order to avoid temperature inversions (tasmax < tasmin), we adjusted dtr and reconstructed tasmin in a subsequent step. As a consequence, co-occurrence of small errors from both dtr and tasmax can accumulate and decrease the performance of extremes in tasmin. Figure 7 shows that the bias-adjusted simulations (d) reproduce the spatial pattern of the RDRS v2.1 reference much better than the original simulation (b) even if there is a cold bias and about half the grid cells values of the original simulation are closer to RDRS v2.1 than the bias-adjusted simulation values (see Fig. 5). We can also see that the RMSE is smaller for the bias-adjusted simulation than for the original. This result is representative of tasmin’s marginal properties in general.

#### Temporal aspect

Because the bias-adjustment method is applied to each day of the year independently, we expect the bias-adjusted simulations to accurately reproduce the RDRS v2.1 annual cycle. The average *IMP* of the amplitude of the annual cycle of maximum temperatures (aca_tasmax) is 93% over all models. There are also large improvements in the RMSE. For the relative amplitude of precipitation (aca_pr), this ratio decreases to 72%. This difference can be explained by a weaker annual cycle in some regions compared to temperature.

**Fig. 8:**
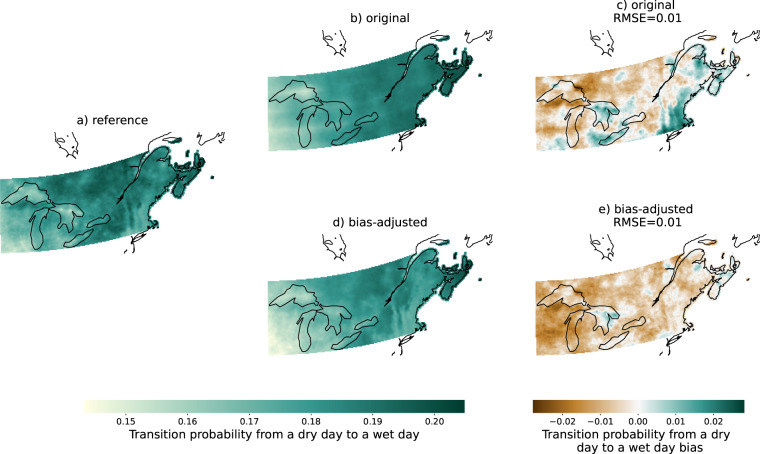
Same as Fig. 6, but for the transition probability from a dry day to a wet day and FGOALS-g3 SSP3-7.0.

On the other hand, the properties measuring sequences of days have not been explicitly corrected, but most of them nonetheless still perform reasonably well compared to the original simulations, with an average *IMP* of 81% for maximum length of warm spell, 73% for maximum length of dry spell, and 85% for wet-wet transition. The property with the lowest *IMP* was the dry-wet transition with 62%. Figure 8 shows that, for this property, there was very little change between the original and bias-adjusted simulations. This might be due to the second pre-processing step of bias-adjustment, which adapts the frequency of dry days. We recommend that users interested in dry-wet transitions use the original simulations directly as the RMSE is already very small.

#### Multivariate aspect

Our bias-adjustment method is univariate, in the sense that each variable is corrected independently. However, the workflow for each variable is not completely independent, as tasmin is reconstructed from tasmax and dtr. This could explain in part the mean *IMP* of 93% for the correlation between tasmax and tasmin. That said, the *IMP* of the correlation between tasmax and pr is also high (89%), even though they were not corrected together. From the RMSE, we can see that the bias-adjustment helps both properties, but the tasmax and tasmin correlation was already well represented in the original simulation.

## Usage Notes

The dataset is available through a THREDDS Data Server (TDS). NetCDF files can be downloaded through the link provided above with the http server access. As the dataset contains many netCDF files with only 4 years of data and one variable, an easier way to access the data is through NcMLs: https://pavics.ouranos.ca/twitcher/ows/proxy/thredds/catalog/datasets/simulations/bias_adjusted/cmip6/ouranos/ESPO-G/ESPO-G6-R2v1.0.0/catalog.html. NcMLs are aggregations of netCDF files that can be accessed using *xarray* (https://docs.xarray.dev)^35^ via the *OPeNDAP* protocol. A general workflow might look like:

1. Select an NcML,
2. Select the OPeNDAP access,
3. Copy-paste the data URL (url) into your access call:

xarray.open_dataset(url, chunks = dict(time = 1460, rlat = 50, rlon = 50))


We recommend using the *xclim* (https://xclim.readthedocs.io)^18^ and *xscen* (https://xscen.readthedocs.io)^36^ packages to perform further analysis on the data, including data validation/quality assurance, computing indicators, climatologies, projected change, and ensemble statistics. Additional examples of data analysis are available on PAVICS (https://pavics.ouranos.ca) and on the ESPO-G GitHub repository (https://github.com/Ouranosinc/ESPO-G). For less technical users, a series of indicators computed from the ESPO-G6-R2 dataset over Quebec can be consulted on the Ouranos *Portraits Climatiques* website (https://portraits.ouranos.ca).

It is important to note that this dataset is meant to serve multiple purposes rather than for a specific application. Other bias-adjustment methods may be better suited for a given use case. It is the responsibility of the user to verify that the dataset best reflects their needs and their region of interest.

### Supplementary information

Supplementary Information

## Data Availability

The code to reproduce the dataset ESPO-G6-R2 dataset and the figures from this paper are available in the release ESPO-G6-R2 v1.0.0 (https://github.com/Ouranosinc/ESPO-G/releases/tag/ESPO-G6-R2v1.0.0) of the ESPO-G GitHub repository (https://github.com/Ouranosinc/ESPO-G). The code works with *xclim* version 0.41.0 and *xscen* version 0.5.13.
