# Supplementary material for: An ensemble of bias-adjusted CMIP6 climate simulations based on a high-resolution North American reanalysis
Source: Sci Data. 2024 Jan 11;11:64. doi: 10.1038/s41597-023-02855-z (PMC10784550; doi:10.1038/s41597-023-02855-z)
Supplement: Supplementary file 1 — Supplementary Information [file 41597_2023_2855_MOESM1_ESM.pdf]

Supplemental Information for *An ensemble of bias-adjusted CMIP6 climate simulations based on a high-resolution North American reanalysis*

Juliette Lavoie, Pascal Bourgault, Trevor James Smith, Travis Logan, Martin Leduc, Louis-Philippe Caron, Sarah Gammon, Marco Braun

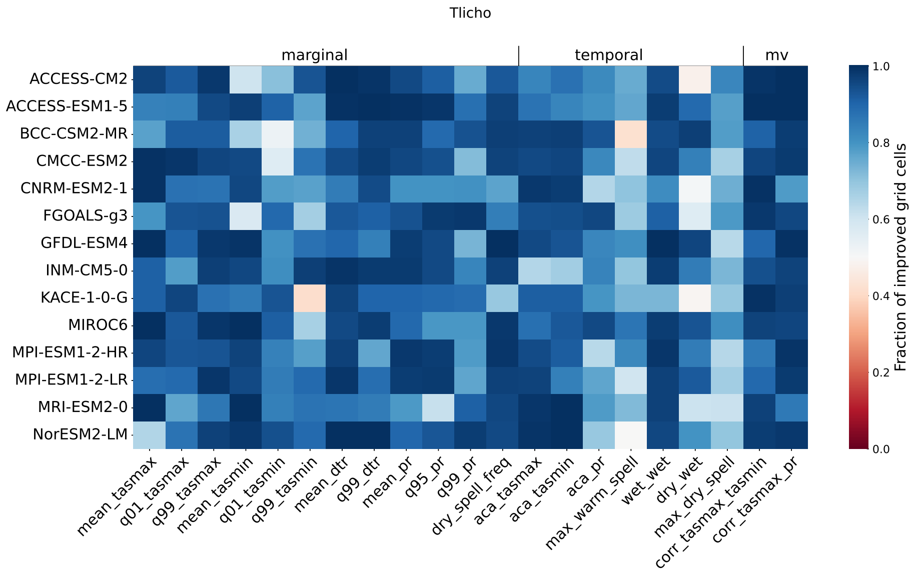

Figure S1: Heatmap of the percentage of improved grid cells (IMP) between the original and the bias-adjusted simulations over the Tlicho region for SSP3-7.0. The columns represent the properties (identified by their short name) while the rows represent the climate models. A value close to 1 means that the bias-adjustment improves the given property over most of the area compared to the original simulation, while a value close to 0 means that the original simulation provides a better performance.

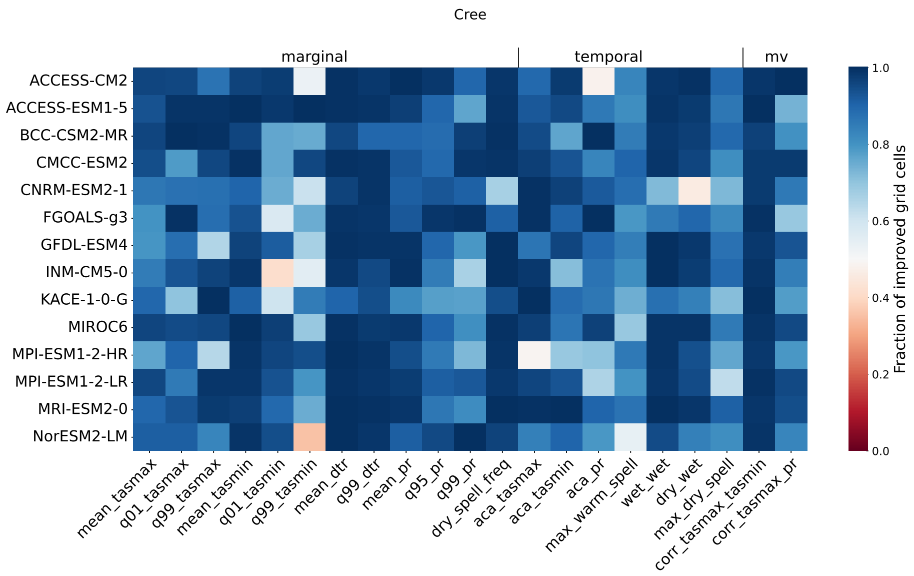

Figure S2: Heatmap of the percentage of improved grid cells (IMP) between the original and the bias-adjusted simulations over the Cree region for SSP3-7.0. The columns represent the properties (identified by their short name) while the rows represent the climate models. A value close to 1 means that the bias-adjustment improves the given property over most of the area compared to the original simulation, while a value close to 0 means that the original simulation provides a better performance.

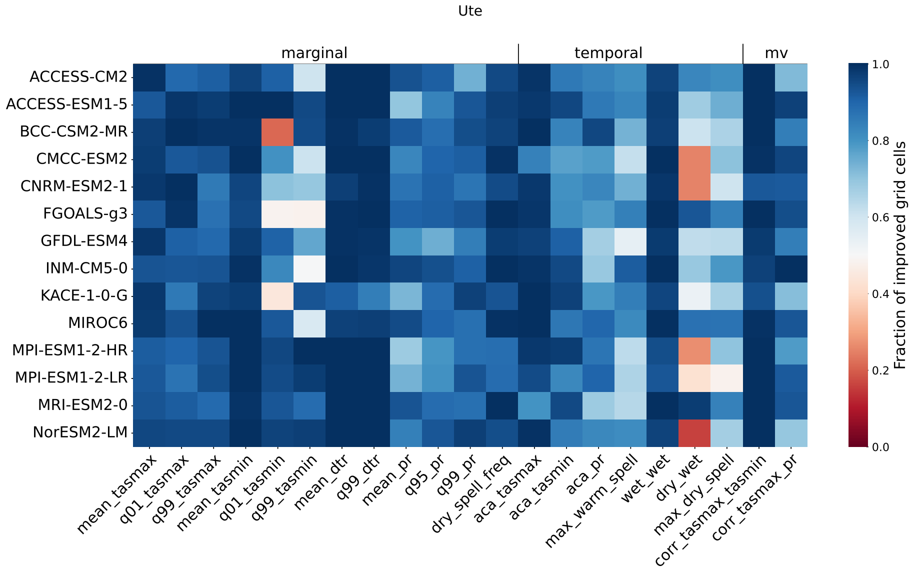

Figure S3: Heatmap of the percentage of improved grid cells (IMP) between the original and the bias-adjusted simulations over the Ute region for SSP3-7.0. The columns represent the properties (identified by their short name) while the rows represent the climate models. A value close to 1 means that the bias-adjustment improves the given property over most of the area compared to the original simulation, while a value close to 0 means that the original simulation provides a better performance.

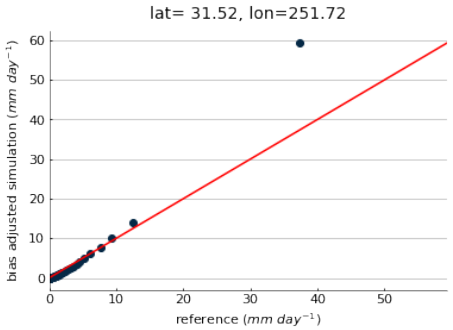

Figure S4: Q-Q plot of the precipitation at one random grid cells for the SSP3-7.0 MIROC6 bias-adjusted simulation and reference regridded on the original GCM grid over the 1981-2010 period. 100 points are plotted, one for each percentile.

Table S1: Each model and ensemble mean RMSE for the the mean daily maximum temperature and each region for SSP3-7.0 between reference and original simulation (OR) and between reference and bias-adjustment simulation (BA).The smaller RMSE is in bold.

**mean_tasmax (°C)**								
	Magtogoek		Tlicho		Ute		Cree	
	OR	BA	OR	BA	OR	BA	OR	BA
ACCESS-CM2	2.37	**0.25**	2.50	**0.45**	3.26	**0.18**	2.50	**0.36**
ACCESS-ESM1-5	2.01	**0.32**	1.42	**0.23**	2.61	**0.27**	2.86	**0.41**
BCC-CSM2-MR	1.42	**0.16**	0.87	**0.21**	2.07	**0.08**	1.63	**0.11**
CMCC-ESM2	2.27	**0.24**	1.97	**0.18**	2.01	**0.08**	1.98	**0.20**
CNRM-ESM2-1	1.76	**0.20**	1.70	**0.11**	3.48	**0.32**	1.57	**0.24**
FGOALS-g3	1.40	**0.43**	1.61	**0.35**	3.19	**0.19**	1.42	**0.31**
GFDL-ESM4	2.24	**0.26**	1.76	**0.11**	2.51	**0.20**	1.61	**0.36**
INM-CM5-0	1.39	**0.32**	1.23	**0.18**	2.12	**0.27**	1.58	**0.34**
KACE-1-0-G	1.59	**0.20**	1.58	**0.51**	2.59	**0.11**	1.92	**0.42**
MIROC6	2.94	**0.33**	3.65	**0.14**	3.58	**0.23**	3.05	**0.40**
MPI-ESM1-2-HR	1.62	**0.10**	1.60	**0.20**	1.53	**0.10**	1.32	**0.33**
MPI-ESM1-2-LR	1.79	**0.13**	1.24	**0.20**	2.25	**0.25**	1.83	**0.11**
MRI-ESM2-0	1.28	**0.27**	3.77	**0.08**	2.75	**0.33**	1.58	**0.27**
NorESM2-LM	1.69	**0.23**	1.24	**0.47**	2.74	**0.21**	1.55	**0.25**
average	1.84	**0.25**	1.87	**0.24**	2.62	**0.20**	1.89	**0.29**

Table S2: Same as Table S1 for the first percentile of daily maximum temperature.

q01_tasmax (°C)								
	Magtogoek		Tlicho		Ute		Cree	
	OR	BA	OR	BA	OR	BA	OR	BA
ACCESS-CM2	5.63	**0.44**	3.85	**0.44**	1.83	**0.43**	2.11	**0.26**
ACCESS-ESM1-5	4.16	**0.70**	3.54	**0.88**	4.08	**0.35**	4.45	**0.57**
BCC-CSM2-MR	7.34	**0.78**	2.79	**0.36**	3.98	**0.26**	3.95	**0.25**
CMCC-ESM2	3.20	**1.20**	3.51	**0.30**	2.58	**0.43**	1.03	**0.41**
CNRM-ESM2-1	3.16	**0.59**	3.42	**0.59**	5.84	**0.55**	3.37	**0.61**
FGOALS-g3	5.35	**1.02**	4.71	**0.96**	3.51	**0.30**	2.97	**0.31**
GFDL-ESM4	1.74	**0.67**	2.57	**0.58**	2.30	**0.38**	1.67	**0.50**
INM-CM5-0	3.23	**0.75**	2.79	**1.07**	3.60	**0.40**	2.54	**0.35**
KACE-1-0-G	4.20	**0.31**	4.68	**0.50**	2.90	**0.74**	2.05	**0.56**
MIROC6	3.42	**0.88**	2.68	**0.48**	2.09	**0.27**	2.42	**0.37**
MPI-ESM1-2-HR	3.99	**0.37**	2.39	**0.44**	1.61	**0.34**	2.08	**0.51**
MPI-ESM1-2-LR	4.40	**0.29**	1.69	**0.35**	1.88	**0.50**	2.20	**0.54**
MRI-ESM2-0	4.60	**0.69**	2.55	**0.72**	2.68	**0.33**	3.74	**0.46**
NorESM2-LM	2.40	**0.62**	2.21	**0.52**	2.74	**0.27**	2.72	**0.64**
average	4.06	**0.67**	3.10	**0.59**	2.97	**0.40**	2.67	**0.45**

Table S3: Same as Table S1 for the 99th percentile of daily maximum temperature .

q99_tasmax (°C)								
	Magtogoek		Tlicho		Ute		Cree	
	OR	BA	OR	BA	OR	BA	OR	BA
ACCESS-CM2	4.42	**0.55**	4.65	**0.41**	3.04	**0.52**	3.69	**1.09**
ACCESS-ESM1-5	6.43	**0.47**	3.41	**0.25**	4.84	**0.44**	6.79	**0.35**
BCC-CSM2-MR	8.91	**0.30**	2.92	**0.33**	6.25	**0.16**	5.55	**0.25**
CMCC-ESM2	4.46	**0.20**	2.72	**0.21**	3.38	**0.33**	3.76	**0.37**
CNRM-ESM2-1	3.40	**0.33**	3.14	**0.37**	2.97	**0.69**	3.08	**0.59**
FGOALS-g3	5.38	**0.61**	3.73	**0.28**	3.24	**0.60**	4.24	**0.82**
GFDL-ESM4	3.44	**0.62**	4.35	**0.37**	2.21	**0.44**	2.56	**0.63**
INM-CM5-0	4.26	**0.46**	4.80	**0.30**	4.74	**0.65**	3.45	**0.55**
KACE-1-0-G	8.28	**0.75**	3.37	**0.58**	6.34	**0.52**	8.68	**0.90**
MIROC6	4.70	**0.44**	6.04	**0.27**	9.94	**0.66**	4.45	**0.97**
MPI-ESM1-2-HR	3.58	**0.36**	2.81	**0.40**	3.27	**0.36**	2.29	**0.75**
MPI-ESM1-2-LR	3.78	**0.29**	4.58	**0.38**	3.34	**0.33**	3.34	**0.30**
MRI-ESM2-0	3.31	**0.38**	3.18	**0.39**	2.69	**0.52**	3.59	**0.59**
NorESM2-LM	4.89	**0.33**	4.61	**0.38**	4.74	**0.55**	3.76	**0.71**
average	4.95	**0.43**	3.88	**0.35**	4.36	**0.48**	4.23	**0.63**

Table S4: Same as Table S1 for the mean of the daily minimum temperature.

**mean_tasmin (°C)**								
	Magtogoek		Tlicho		Ute		Cree	
	OR	BA	OR	BA	OR	BA	OR	BA
ACCESS-CM2	1.84	**0.20**	1.72	**0.48**	2.52	**0.20**	2.07	**0.19**
ACCESS-ESM1-5	3.77	**0.29**	3.60	**0.33**	5.68	**0.21**	5.03	**0.37**
BCC-CSM2-MR	2.29	**0.20**	1.53	**0.30**	2.42	**0.10**	1.31	**0.11**
CMCC-ESM2	2.23	**0.27**	2.31	**0.22**	4.23	**0.11**	4.02	**0.23**
CNRM-ESM2-1	2.72	**0.25**	2.37	**0.25**	1.69	**0.27**	1.22	**0.22**
FGOALS-g3	1.77	**0.45**	1.84	**0.46**	1.87	**0.24**	1.55	**0.28**
GFDL-ESM4	1.30	**0.25**	2.53	**0.20**	1.91	**0.14**	1.96	**0.28**
INM-CM5-0	2.09	**0.40**	2.73	**0.16**	4.33	**0.17**	3.02	**0.34**
KACE-1-0-G	1.61	**0.21**	1.99	**0.58**	2.31	**0.15**	1.58	**0.29**
MIROC6	3.95	**0.34**	4.70	**0.18**	5.42	**0.15**	4.74	**0.28**
MPI-ESM1-2-HR	1.73	**0.11**	2.66	**0.30**	3.67	**0.09**	2.73	**0.30**
MPI-ESM1-2-LR	1.76	**0.11**	2.24	**0.30**	3.77	**0.10**	2.44	**0.14**
MRI-ESM2-0	1.71	**0.23**	3.78	**0.20**	3.33	**0.23**	4.02	**0.20**
NorESM2-LM	2.58	**0.22**	3.68	**0.45**	6.43	**0.20**	3.45	**0.25**
average	2.24	**0.25**	2.69	**0.31**	3.54	**0.17**	2.80	**0.25**

Table S5: Same as Table S1 for the first percentile of daily minimum temperature.

**q01_tasmin (°C)**								
	Magtogoek		Tlicho		Ute		Cree	
	OR	BA	OR	BA	OR	BA	OR	BA
ACCESS-CM2	5.73	**0.95**	3.35	**1.33**	7.54	**2.48**	7.58	**1.41**
ACCESS-ESM1-5	7.36	**1.65**	6.51	**1.74**	10.89	**2.01**	9.68	**1.63**
BCC-CSM2-MR	9.01	**1.57**	2.97	**1.80**	**3.43**	4.11	4.08	**1.71**
CMCC-ESM2	5.31	**3.51**	3.51	**1.40**	6.61	**2.96**	3.80	**2.66**
CNRM-ESM2-1	5.23	**1.17**	3.36	**1.35**	3.44	**2.28**	2.56	**1.44**
FGOALS-g3	8.22	**2.39**	4.34	**1.32**	**3.02**	3.04	3.11	**2.27**
GFDL-ESM4	3.37	**0.96**	2.88	**0.90**	5.74	**1.43**	5.94	**1.26**
INM-CM5-0	6.65	**3.24**	4.08	**1.32**	6.86	**3.65**	**3.93**	4.14
KACE-1-0-G	8.11	**2.62**	6.03	**1.11**	3.32	**2.96**	3.26	**1.96**
MIROC6	6.73	**2.25**	5.44	**1.81**	7.27	**2.00**	7.48	**1.32**
MPI-ESM1-2-HR	4.78	**1.16**	3.02	**0.91**	6.06	**1.61**	6.84	**2.43**
MPI-ESM1-2-LR	4.86	**1.22**	2.66	**0.99**	5.83	**1.45**	6.24	**2.01**
MRI-ESM2-0	5.06	**1.42**	3.48	**1.43**	8.00	**2.59**	7.86	**2.76**
NorESM2-LM	4.30	**1.67**	4.99	**1.31**	9.47	**2.24**	7.51	**1.28**
average	6.05	**1.84**	4.04	**1.34**	6.25	**2.49**	5.71	**2.02**

Table S6: Same as Table S1 for the 99th percentile of daily minimum temperature.

**q99_tasmin (°C)**								
	Magtogoek		Tlicho		Ute		Cree	
	OR	BA	OR	BA	OR	BA	OR	BA
ACCESS-CM2	2.11	**0.67**	3.29	**0.94**	2.07	**1.36**	1.53	**1.05**
ACCESS-ESM1-5	3.74	**1.01**	2.00	**0.45**	4.58	**1.04**	4.63	**1.15**
BCC-CSM2-MR	1.76	**1.53**	2.82	**1.76**	3.23	**0.57**	1.99	**1.07**
CMCC-ESM2	2.46	**0.73**	2.03	**0.44**	2.93	**1.62**	3.63	**0.95**
CNRM-ESM2-1	1.70	**1.07**	1.75	**0.53**	2.73	**1.35**	1.49	**0.91**
FGOALS-g3	2.36	**1.57**	2.25	**1.19**	1.66	**1.24**	1.94	**1.16**
GFDL-ESM4	0.98	**0.64**	2.68	**0.60**	2.40	**0.81**	1.40	**0.62**
INM-CM5-0	1.21	**0.92**	2.64	**0.42**	2.47	**1.63**	1.52	**0.98**
KACE-1-0-G	4.38	**1.29**	1.96	**1.71**	3.52	**0.68**	3.37	**1.09**
MIROC6	1.74	**1.61**	2.73	**1.10**	4.47	**2.22**	2.20	**1.63**
MPI-ESM1-2-HR	2.63	**0.71**	1.48	**0.37**	6.12	**0.64**	2.71	**0.51**
MPI-ESM1-2-LR	1.96	**0.68**	1.98	**0.44**	5.39	**0.62**	1.22	**0.52**
MRI-ESM2-0	2.12	**0.52**	1.52	**0.40**	2.45	**0.71**	1.01	**0.51**
NorESM2-LM	2.99	**1.05**	2.95	**1.31**	5.16	**1.58**	**1.79**	1.91
average	2.30	**1.00**	2.29	**0.83**	3.51	**1.15**	2.17	**1.01**

Table S7: Same as Table S1 for mean of the daily temperature range.

**mean_dtr (°C)**								
	Magtogoek		Tlicho		Ute		Cree	
	OR	BA	OR	BA	OR	BA	OR	BA
ACCESS-CM2	2.76	**0.09**	2.93	**0.06**	4.70	**0.17**	4.03	**0.24**
ACCESS-ESM1-5	3.34	**0.07**	3.46	**0.11**	3.93	**0.10**	2.84	**0.11**
BCC-CSM2-MR	2.77	**0.07**	1.43	**0.11**	2.71	**0.10**	1.89	**0.08**
CMCC-ESM2	2.39	**0.07**	1.19	**0.06**	4.13	**0.12**	2.97	**0.09**
CNRM-ESM2-1	2.41	**0.08**	1.36	**0.18**	2.76	**0.11**	1.51	**0.07**
FGOALS-g3	2.82	**0.05**	1.64	**0.13**	4.01	**0.11**	2.40	**0.08**
GFDL-ESM4	2.89	**0.06**	1.32	**0.15**	3.61	**0.11**	2.87	**0.12**
INM-CM5-0	2.93	**0.10**	2.18	**0.06**	3.65	**0.16**	2.33	**0.08**
KACE-1-0-G	2.42	**0.17**	1.45	**0.15**	1.58	**0.17**	1.18	**0.19**
MIROC6	2.49	**0.07**	1.79	**0.06**	2.90	**0.17**	2.51	**0.16**
MPI-ESM1-2-HR	2.89	**0.06**	1.56	**0.11**	3.77	**0.12**	3.05	**0.08**
MPI-ESM1-2-LR	3.03	**0.15**	1.85	**0.11**	4.15	**0.23**	3.26	**0.12**
MRI-ESM2-0	2.60	**0.10**	1.48	**0.20**	5.40	**0.14**	4.07	**0.13**
NorESM2-LM	2.53	**0.06**	3.06	**0.06**	4.59	**0.11**	3.20	**0.07**
average	2.73	**0.09**	1.91	**0.11**	3.71	**0.14**	2.72	**0.12**

Table S8: Same as Table S1 for the 99th percentile of tha daily temperature range.

**q99_dtr (°C)**								
	Magtogoek		Tlicho		Ute		Cree	
	OR	BA	OR	BA	OR	BA	OR	BA
ACCESS-CM2	4.18	**0.32**	6.17	**0.31**	5.77	**0.31**	6.55	**0.28**
ACCESS-ESM1-5	5.59	**0.26**	7.27	**0.43**	5.34	**0.19**	4.16	**0.20**
BCC-CSM2-MR	4.92	**0.35**	3.56	**0.35**	2.57	**0.22**	1.97	**0.21**
CMCC-ESM2	3.69	**0.29**	2.29	**0.22**	6.13	**0.21**	4.93	**0.31**
CNRM-ESM2-1	4.66	**0.30**	3.20	**0.30**	4.52	**0.24**	3.64	**0.22**
FGOALS-g3	4.00	**0.28**	2.20	**0.31**	6.97	**0.24**	4.20	**0.36**
GFDL-ESM4	4.16	**0.30**	1.74	**0.38**	4.98	**0.22**	4.05	**0.17**
INM-CM5-0	4.16	**0.43**	3.87	**0.27**	3.65	**0.29**	2.50	**0.37**
KACE-1-0-G	3.91	**0.43**	2.36	**0.47**	1.76	**0.34**	2.16	**0.40**
MIROC6	4.02	**0.34**	3.06	**0.24**	3.72	**0.44**	3.52	**0.30**
MPI-ESM1-2-HR	4.20	**0.24**	1.81	**0.45**	6.02	**0.46**	5.18	**0.34**
MPI-ESM1-2-LR	4.11	**0.41**	1.50	**0.25**	6.60	**0.34**	5.42	**0.39**
MRI-ESM2-0	3.90	**0.30**	2.03	**0.39**	8.03	**0.19**	6.66	**0.22**
NorESM2-LM	4.31	**0.29**	5.40	**0.38**	7.42	**0.22**	4.47	**0.29**
average	4.27	**0.33**	3.32	**0.34**	5.25	**0.28**	4.24	**0.29**

Table S9: Same as Table S1 for the mean of the precipitation.

**mean_pr (mm d^{-1})**								
	Magtogoek		Tlicho		Ute		Cree	
	OR	BA	OR	BA	OR	BA	OR	BA
ACCESS-CM2	0.42	**0.08**	0.23	**0.04**	0.59	**0.09**	0.76	**0.05**
ACCESS-ESM1-5	0.52	**0.10**	0.52	**0.03**	0.36	**0.12**	0.57	**0.05**
BCC-CSM2-MR	0.30	**0.07**	0.27	**0.03**	0.42	**0.08**	0.31	**0.05**
CMCC-ESM2	0.27	**0.06**	0.24	**0.04**	0.75	**0.11**	0.37	**0.05**
CNRM-ESM2-1	0.35	**0.07**	0.30	**0.06**	0.46	**0.09**	0.35	**0.05**
FGOALS-g3	0.61	**0.07**	0.28	**0.04**	0.98	**0.12**	0.40	**0.04**
GFDL-ESM4	0.50	**0.06**	0.45	**0.05**	0.35	**0.08**	0.53	**0.04**
INM-CM5-0	0.48	**0.06**	0.37	**0.04**	0.80	**0.07**	0.54	**0.05**
KACE-1-0-G	0.37	**0.10**	0.19	**0.03**	0.32	**0.11**	0.32	**0.09**
MIROC6	0.46	**0.07**	0.25	**0.04**	0.67	**0.07**	0.62	**0.05**
MPI-ESM1-2-HR	0.47	**0.06**	0.35	**0.03**	0.33	**0.12**	0.38	**0.06**
MPI-ESM1-2-LR	0.40	**0.07**	0.48	**0.03**	0.49	**0.18**	0.45	**0.07**
MRI-ESM2-0	0.48	**0.07**	0.20	**0.05**	0.87	**0.10**	0.56	**0.05**
NorESM2-LM	0.40	**0.10**	0.20	**0.04**	0.47	**0.10**	0.36	**0.07**
average	0.43	**0.07**	0.31	**0.04**	0.56	**0.10**	0.47	**0.05**

Table S10: Same as Table S1 for the 95th percentile of precipitation.

**q95_pr (mm d^{-1})**								
	Magtogoek		Tlicho		Ute		Cree	
	OR	BA	OR	BA	OR	BA	OR	BA
ACCESS-CM2	1.57	**0.45**	0.93	**0.24**	2.30	**0.44**	3.05	**0.25**
ACCESS-ESM1-5	1.55	**0.51**	1.78	**0.21**	1.93	**0.53**	1.91	**0.25**
BCC-CSM2-MR	2.38	**0.41**	1.28	**0.14**	2.81	**0.56**	1.61	**0.28**
CMCC-ESM2	1.74	**0.37**	0.89	**0.17**	2.85	**0.58**	1.53	**0.27**
CNRM-ESM2-1	1.47	**0.36**	1.19	**0.28**	2.10	**0.37**	1.58	**0.30**
FGOALS-g3	3.83	**0.34**	1.54	**0.23**	4.31	**0.65**	2.91	**0.27**
GFDL-ESM4	1.70	**0.36**	1.27	**0.28**	1.69	**0.53**	1.60	**0.26**
INM-CM5-0	2.08	**0.36**	1.22	**0.19**	2.80	**0.36**	1.23	**0.27**
KACE-1-0-G	1.71	**0.47**	0.80	**0.18**	2.30	**0.63**	1.79	**0.48**
MIROC6	1.62	**0.35**	0.81	**0.22**	2.38	**0.34**	1.73	**0.27**
MPI-ESM1-2-HR	1.93	**0.40**	1.46	**0.17**	2.01	**0.54**	1.45	**0.34**
MPI-ESM1-2-LR	1.90	**0.36**	1.78	**0.17**	2.36	**0.75**	1.72	**0.31**
MRI-ESM2-0	1.64	**0.38**	0.65	**0.28**	2.74	**0.49**	1.71	**0.28**
NorESM2-LM	2.47	**0.38**	0.98	**0.18**	3.68	**0.48**	2.23	**0.28**
average	1.97	**0.39**	1.18	**0.21**	2.59	**0.52**	1.86	**0.29**

Table S11: Same as Table S1 for the 99th percentile of precipitation.

**q99_pr (mm d^{-1})**								
	Magtogoek		Tlicho		Ute		Cree	
	OR	BA	OR	BA	OR	BA	OR	BA
ACCESS-CM2	3.02	**1.19**	1.92	**0.77**	4.24	**1.23**	5.30	**0.85**
ACCESS-ESM1-5	3.64	**1.34**	2.60	**0.58**	6.86	**1.56**	2.80	**0.98**
BCC-CSM2-MR	4.94	**1.06**	4.16	**0.73**	6.84	**1.11**	5.48	**1.06**
CMCC-ESM2	4.12	**1.18**	1.61	**0.67**	8.66	**1.59**	4.93	**0.87**
CNRM-ESM2-1	3.99	**1.10**	2.15	**0.86**	5.59	**1.18**	4.08	**1.03**
FGOALS-g3	8.45	**1.09**	5.63	**0.75**	10.92	**1.73**	9.59	**1.01**
GFDL-ESM4	3.51	**1.13**	1.90	**0.84**	3.86	**1.13**	2.57	**0.77**
INM-CM5-0	3.70	**1.08**	2.55	**0.79**	5.77	**1.16**	2.59	**0.97**
KACE-1-0-G	4.16	**1.12**	2.71	**0.67**	7.03	**1.51**	5.25	**1.44**
MIROC6	2.65	**1.28**	2.30	**0.68**	5.03	**0.96**	2.64	**0.88**
MPI-ESM1-2-HR	3.86	**1.18**	1.78	**0.65**	6.31	**1.60**	2.53	**0.88**
MPI-ESM1-2-LR	5.47	**1.09**	2.81	**0.65**	8.72	**2.10**	3.74	**0.99**
MRI-ESM2-0	4.11	**1.23**	3.00	**0.87**	5.87	**1.24**	2.67	**0.88**
NorESM2-LM	8.03	**1.23**	4.14	**0.76**	12.87	**1.37**	8.44	**0.88**
average	4.55	**1.16**	2.81	**0.73**	7.04	**1.39**	4.47	**0.96**

Table S12: Same as Table S1 for dry spell frequency.

**dry_spell_freq**								
	Magtogoek		Tlicho		Ute		Cree	
	OR	BA	OR	BA	OR	BA	OR	BA
ACCESS-CM2	0.07	**0.02**	0.05	**0.02**	0.13	**0.01**	0.13	**0.01**
ACCESS-ESM1-5	0.11	**0.01**	0.13	**0.01**	0.13	**0.02**	0.14	**0.01**
BCC-CSM2-MR	0.06	**0.02**	0.09	**0.01**	0.09	**0.01**	0.11	**0.01**
CMCC-ESM2	0.05	**0.02**	0.07	**0.01**	0.20	**0.01**	0.12	**0.01**
CNRM-ESM2-1	0.05	**0.02**	0.06	**0.02**	0.09	**0.01**	0.06	**0.01**
FGOALS-g3	0.07	**0.02**	0.08	**0.01**	0.22	**0.01**	0.11	**0.01**
GFDL-ESM4	0.11	**0.01**	0.14	**0.01**	0.09	**0.01**	0.13	**0.01**
INM-CM5-0	0.11	**0.01**	0.11	**0.01**	0.23	**0.01**	0.18	**0.00**
KACE-1-0-G	0.05	**0.03**	0.04	**0.01**	0.08	**0.02**	0.06	**0.01**
MIROC6	0.08	**0.01**	0.09	**0.01**	0.18	**0.01**	0.16	**0.01**
MPI-ESM1-2-HR	0.07	**0.01**	0.09	**0.01**	0.07	**0.02**	0.08	**0.01**
MPI-ESM1-2-LR	0.08	**0.01**	0.13	**0.01**	0.13	**0.03**	0.12	**0.01**
MRI-ESM2-0	0.10	**0.01**	0.07	**0.01**	0.20	**0.01**	0.15	**0.01**
NorESM2-LM	0.05	**0.02**	0.08	**0.01**	0.10	**0.02**	0.09	**0.01**
average	0.07	**0.02**	0.09	**0.01**	0.14	**0.01**	0.12	**0.01**

Table S13: Same as Table S1 for amplitude of the annual cycle of the daily maximum temperature.

**aca_tasmax (°C)**								
	Magtogoek		Tlicho		Ute		Cree	
	OR	BA	OR	BA	OR	BA	OR	BA
ACCESS-CM2	7.14	**0.23**	3.85	**0.75**	5.21	**0.35**	2.32	**0.55**
ACCESS-ESM1-5	5.43	**0.32**	3.77	**0.66**	4.51	**0.36**	2.95	**0.60**
BCC-CSM2-MR	8.94	**0.22**	8.03	**1.56**	8.64	**0.27**	4.39	**0.80**
CMCC-ESM2	4.86	**0.74**	4.36	**0.49**	2.88	**0.60**	3.88	**0.45**
CNRM-ESM2-1	3.95	**0.33**	4.68	**0.29**	3.46	**0.46**	4.97	**0.33**
FGOALS-g3	7.97	**0.44**	4.31	**0.65**	5.42	**0.52**	5.79	**1.00**
GFDL-ESM4	2.52	**0.49**	6.02	**0.96**	4.03	**0.41**	1.80	**0.52**
INM-CM5-0	4.00	**0.60**	2.92	**1.33**	4.44	**0.24**	4.30	**0.42**
KACE-1-0-G	9.77	**0.80**	5.78	**0.73**	11.05	**0.35**	9.76	**1.67**
MIROC6	3.66	**0.28**	4.57	**0.93**	9.80	**0.77**	3.89	**0.89**
MPI-ESM1-2-HR	4.94	**0.27**	3.48	**0.48**	4.20	**0.48**	1.52	**0.83**
MPI-ESM1-2-LR	4.19	**0.32**	4.84	**0.67**	3.79	**0.30**	2.80	**0.65**
MRI-ESM2-0	4.50	**0.63**	5.29	**0.82**	2.38	**0.69**	5.74	**0.35**
NorESM2-LM	5.04	**0.29**	8.67	**0.87**	5.60	**0.34**	3.18	**0.63**
average	5.49	**0.42**	5.04	**0.80**	5.39	**0.44**	4.09	**0.69**

Table S14: Same as Table S1 for the amplitude of the annual cycle of the daily minimum temperature.

**aca_tasmin (°C)**								
	Magtogoek		Tlicho		Ute		Cree	
	OR	BA	OR	BA	OR	BA	OR	BA
ACCESS-CM2	4.84	**0.37**	2.69	**0.63**	2.68	**0.57**	3.63	**0.49**
ACCESS-ESM1-5	4.10	**0.53**	2.75	**0.54**	2.96	**0.38**	2.38	**0.26**
BCC-CSM2-MR	5.15	**0.31**	5.38	**1.16**	2.55	**0.52**	2.04	**0.79**
CMCC-ESM2	5.09	**1.01**	4.42	**0.32**	2.36	**0.66**	3.33	**0.60**
CNRM-ESM2-1	3.46	**0.32**	4.36	**0.29**	1.80	**0.41**	3.04	**0.46**
FGOALS-g3	6.68	**0.40**	3.39	**0.63**	2.06	**0.45**	3.14	**0.88**
GFDL-ESM4	2.09	**0.58**	4.96	**0.83**	2.41	**0.41**	3.26	**0.39**
INM-CM5-0	5.35	**0.94**	2.56	**1.38**	3.50	**0.49**	2.11	**0.98**
KACE-1-0-G	7.97	**0.89**	6.14	**0.60**	3.62	**0.59**	4.11	**1.27**
MIROC6	2.46	**0.60**	3.74	**0.82**	2.53	**0.54**	2.37	**0.67**
MPI-ESM1-2-HR	5.74	**0.36**	2.59	**0.40**	3.49	**0.55**	1.51	**0.70**
MPI-ESM1-2-LR	5.45	**0.31**	2.24	**0.48**	2.68	**0.68**	2.51	**0.47**
MRI-ESM2-0	3.93	**0.55**	6.80	**0.94**	4.08	**0.55**	7.04	**0.50**
NorESM2-LM	4.29	**0.37**	8.25	**0.81**	2.15	**0.47**	3.47	**1.00**
average	4.76	**0.54**	4.31	**0.70**	2.78	**0.52**	3.14	**0.68**

Table S15: Same as Table S1 for relative amplitude of the annual cycle of the precipitation.

**aca_pr (%)**								
	Magtogoek		Tlicho		Ute		Cree	
	OR	BA	OR	BA	OR	BA	OR	BA
ACCESS-CM2	26.33	**9.82**	42.07	**16.71**	41.58	**13.82**	25.81	**21.68**
ACCESS-ESM1-5	19.07	**9.78**	40.81	**12.65**	38.08	**12.34**	38.54	**15.57**
BCC-CSM2-MR	24.05	**11.13**	68.26	**18.22**	65.75	**12.73**	94.04	**15.21**
CMCC-ESM2	17.11	**8.90**	30.56	**10.73**	31.99	**12.34**	36.70	**15.01**
CNRM-ESM2-1	19.69	**12.65**	34.35	**17.36**	38.56	**14.56**	62.83	**27.60**
FGOALS-g3	19.74	**8.92**	73.05	**12.18**	34.69	**11.59**	118.25	**17.75**
GFDL-ESM4	17.53	**11.05**	32.60	**9.28**	27.94	**12.32**	45.78	**14.53**
INM-CM5-0	15.79	**7.99**	36.21	**11.30**	35.71	**17.18**	52.63	**17.95**
KACE-1-0-G	19.32	**11.38**	32.20	**11.20**	41.49	**13.85**	47.08	**16.28**
MIROC6	20.64	**7.34**	55.32	**12.79**	40.47	**11.60**	65.83	**17.99**
MPI-ESM1-2-HR	19.38	**10.64**	25.85	**12.87**	49.53	**15.44**	25.56	**13.74**
MPI-ESM1-2-LR	21.15	**11.94**	35.03	**10.57**	57.48	**15.75**	33.64	**18.11**
MRI-ESM2-0	21.03	**8.05**	39.20	**15.87**	28.85	**16.13**	42.92	**20.31**
NorESM2-LM	16.82	**10.27**	34.64	**14.64**	36.38	**10.88**	35.87	**15.56**
average	19.83	**9.99**	41.44	**13.31**	40.61	**13.61**	51.82	**17.66**

Table S16: Same as Table S1 for the maximum length of the warm spell.

**max_warm_spell (days)**								
	Magtogoek		Tlicho		Ute		Cree	
	OR	BA	OR	BA	OR	BA	OR	BA
ACCESS-CM2	14.56	**11.71**	**6.78**	7.56	12.47	**8.67**	15.92	**11.16**
ACCESS-ESM1-5	16.97	**14.91**	**7.38**	7.84	20.26	**16.88**	20.50	**19.60**
BCC-CSM2-MR	17.21	**15.32**	**7.94**	10.33	10.03	**8.84**	7.76	**7.56**
CMCC-ESM2	14.94	**11.73**	**10.87**	12.26	14.96	**12.91**	12.65	**9.27**
CNRM-ESM2-1	18.88	**16.99**	11.51	**11.18**	22.75	**18.51**	12.72	**11.55**
FGOALS-g3	13.97	**11.51**	**10.74**	10.88	17.19	**12.16**	12.28	**8.36**
GFDL-ESM4	16.23	**14.11**	9.24	**8.78**	**14.82**	18.83	10.53	**9.15**
INM-CM5-0	15.89	**13.63**	**11.08**	11.29	26.69	**13.46**	14.06	**8.87**
KACE-1-0-G	14.43	**12.29**	**9.38**	9.55	16.99	**12.90**	14.59	**9.46**
MIROC6	14.17	**13.26**	20.40	**18.95**	9.25	**8.70**	11.38	**8.92**
MPI-ESM1-2-HR	15.51	**13.72**	14.74	**13.92**	23.09	**20.64**	18.08	**14.88**
MPI-ESM1-2-LR	15.80	**14.03**	14.49	**12.70**	18.36	**16.02**	18.47	**16.57**
MRI-ESM2-0	14.27	**11.69**	8.82	**7.46**	**11.48**	12.51	16.68	**12.85**
NorESM2-LM	19.39	**15.13**	**6.59**	9.25	11.78	**8.88**	**8.11**	8.53
average	15.87	**13.57**	**10.71**	10.85	16.44	**13.57**	13.84	**11.20**

Table S17: Same as Table S1 for wet-wet transition.

**wet_wet**								
	Magtogoek		Tlicho		Ute		Cree	
	OR	BA	OR	BA	OR	BA	OR	BA
ACCESS-CM2	0.06	**0.02**	0.05	**0.01**	0.10	**0.01**	0.09	**0.01**
ACCESS-ESM1-5	0.10	**0.01**	0.12	**0.01**	0.11	**0.01**	0.10	**0.01**
BCC-CSM2-MR	0.05	**0.02**	0.07	**0.01**	0.07	**0.01**	0.07	**0.01**
CMCC-ESM2	0.05	**0.02**	0.05	**0.01**	0.20	**0.01**	0.09	**0.01**
CNRM-ESM2-1	0.05	**0.01**	0.05	**0.01**	0.09	**0.01**	0.05	**0.01**
FGOALS-g3	0.06	**0.02**	0.07	**0.01**	0.20	**0.01**	0.08	**0.01**
GFDL-ESM4	0.09	**0.01**	0.12	**0.01**	0.08	**0.01**	0.10	**0.00**
INM-CM5-0	0.10	**0.01**	0.10	**0.01**	0.22	**0.01**	0.14	**0.01**
KACE-1-0-G	0.05	**0.02**	0.04	**0.01**	0.07	**0.01**	0.05	**0.01**
MIROC6	0.07	**0.01**	0.07	**0.01**	0.15	**0.01**	0.12	**0.01**
MPI-ESM1-2-HR	0.06	**0.01**	0.07	**0.01**	0.06	**0.01**	0.06	**0.01**
MPI-ESM1-2-LR	0.07	**0.02**	0.11	**0.01**	0.12	**0.01**	0.09	**0.01**
MRI-ESM2-0	0.09	**0.02**	0.07	**0.01**	0.17	**0.01**	0.11	**0.01**
NorESM2-LM	0.05	**0.03**	0.07	**0.01**	0.09	**0.01**	0.07	**0.01**
average	0.07	**0.02**	0.08	**0.01**	0.12	**0.01**	0.09	**0.01**

Table S18: Same as Table S1 for the dry-wet transition.

**dry_wet**								
	Magtogoek		Tlicho		Ute		Cree	
	OR	BA	OR	BA	OR	BA	OR	BA
ACCESS-CM2	0.01	**0.01**	0.01	**0.01**	0.03	**0.01**	0.04	**0.01**
ACCESS-ESM1-5	0.01	**0.01**	0.02	**0.01**	0.02	**0.01**	0.04	**0.01**
BCC-CSM2-MR	0.02	**0.01**	0.02	**0.00**	0.02	**0.01**	0.04	**0.01**
CMCC-ESM2	0.01	**0.01**	0.02	**0.00**	**0.01**	0.01	0.03	**0.01**
CNRM-ESM2-1	**0.01**	0.01	0.01	**0.01**	0.02	**0.01**	0.01	**0.01**
FGOALS-g3	**0.01**	0.01	0.01	**0.01**	0.03	**0.01**	0.04	**0.01**
GFDL-ESM4	0.02	**0.01**	0.03	**0.00**	0.02	**0.01**	0.04	**0.01**
INM-CM5-0	0.01	**0.01**	0.02	**0.01**	0.02	**0.01**	0.05	**0.01**
KACE-1-0-G	**0.01**	0.01	0.01	**0.01**	0.02	**0.01**	0.02	**0.01**
MIROC6	0.01	**0.01**	0.02	**0.00**	0.03	**0.01**	0.05	**0.01**
MPI-ESM1-2-HR	0.01	**0.01**	0.02	**0.01**	**0.01**	0.01	0.03	**0.01**
MPI-ESM1-2-LR	0.01	**0.00**	0.02	**0.01**	**0.02**	0.02	0.03	**0.01**
MRI-ESM2-0	0.01	**0.01**	0.01	**0.01**	0.03	**0.01**	0.04	**0.01**
NorESM2-LM	0.01	**0.01**	0.02	**0.00**	**0.01**	0.02	0.02	**0.01**
average	0.01	**0.01**	0.02	**0.01**	0.02	**0.01**	0.03	**0.01**

Table S19: Same as Table S1 for the maximum length of dry spell.

**max_dry_spell (days)**								
	Magtogoek		Tlicho		Ute		Cree	
	OR	BA	OR	BA	OR	BA	OR	BA
ACCESS-CM2	**6.87**	6.98	17.24	**15.93**	25.93	**14.98**	26.35	**15.86**
ACCESS-ESM1-5	7.94	**7.13**	17.15	**14.63**	21.16	**13.80**	26.68	**13.90**
BCC-CSM2-MR	7.66	**7.28**	20.87	**14.02**	19.58	**14.65**	27.74	**15.00**
CMCC-ESM2	7.32	**7.28**	16.76	**15.87**	21.20	**18.19**	23.80	**15.77**
CNRM-ESM2-1	**8.36**	9.22	**20.01**	20.01	**15.74**	18.17	**16.00**	16.57
FGOALS-g3	7.86	**6.61**	18.79	**13.93**	26.80	**15.62**	25.06	**15.53**
GFDL-ESM4	8.39	**6.78**	21.67	**16.70**	17.76	**15.72**	25.03	**12.25**
INM-CM5-0	7.72	**6.65**	19.66	**13.62**	28.94	**14.63**	30.05	**14.71**
KACE-1-0-G	**7.34**	8.72	15.93	**14.47**	21.45	**18.15**	18.48	**17.46**
MIROC6	7.97	**7.04**	21.45	**14.53**	27.61	**14.65**	28.66	**16.98**
MPI-ESM1-2-HR	7.43	**6.73**	16.61	**14.47**	**21.73**	22.19	21.60	**19.12**
MPI-ESM1-2-LR	**7.41**	7.42	22.11	**15.34**	30.63	**29.87**	23.38	**20.36**
MRI-ESM2-0	8.64	**7.08**	15.75	**14.37**	27.91	**14.46**	28.53	**16.71**
NorESM2-LM	6.41	**6.33**	16.11	**15.45**	18.62	**15.32**	21.96	**16.04**
average	7.67	**7.23**	18.58	**15.24**	23.22	**17.17**	24.52	**16.16**

Table S20: Same as Table S1 for correlation between daily maximum temperature and daily minimum temperature.

**corr_tasmax_tasmin**								
	Magtogoek		Tlicho		Ute		Cree	
	OR	BA	OR	BA	OR	BA	OR	BA
ACCESS-CM2	0.03	**0.01**	0.02	**0.00**	0.05	**0.01**	0.03	**0.00**
ACCESS-ESM1-5	0.03	**0.00**	0.02	**0.00**	0.05	**0.01**	0.04	**0.00**
BCC-CSM2-MR	0.02	**0.00**	0.01	**0.00**	0.05	**0.01**	0.03	**0.01**
CMCC-ESM2	0.02	**0.00**	0.01	**0.00**	0.04	**0.01**	0.03	**0.00**
CNRM-ESM2-1	0.03	**0.00**	0.02	**0.00**	0.04	**0.01**	0.03	**0.00**
FGOALS-g3	0.03	**0.00**	0.02	**0.00**	0.06	**0.01**	0.04	**0.01**
GFDL-ESM4	0.03	**0.00**	0.01	**0.00**	0.04	**0.00**	0.02	**0.00**
INM-CM5-0	0.04	**0.01**	0.02	**0.00**	0.05	**0.01**	0.03	**0.00**
KACE-1-0-G	0.03	**0.01**	0.02	**0.00**	0.04	**0.01**	0.03	**0.01**
MIROC6	0.02	**0.00**	0.01	**0.00**	0.05	**0.01**	0.03	**0.00**
MPI-ESM1-2-HR	0.03	**0.00**	0.01	**0.00**	0.05	**0.01**	0.03	**0.00**
MPI-ESM1-2-LR	0.03	**0.00**	0.01	**0.00**	0.05	**0.00**	0.03	**0.00**
MRI-ESM2-0	0.03	**0.00**	0.01	**0.00**	0.05	**0.00**	0.03	**0.00**
NorESM2-LM	0.03	**0.00**	0.02	**0.00**	0.07	**0.01**	0.04	**0.01**
average	0.03	**0.00**	0.01	**0.00**	0.05	**0.01**	0.03	**0.00**

Table S21: Same as Table S1 for the correlation between the daily maximum temperature and precipitation.

**corr_tasmax_pr**								
	Magtogoek		Tlicho		Ute		Cree	
	OR	BA	OR	BA	OR	BA	OR	BA
ACCESS-CM2	0.30	**0.15**	0.38	**0.09**	0.11	**0.05**	0.23	**0.08**
ACCESS-ESM1-5	0.17	**0.11**	0.45	**0.07**	0.20	**0.06**	0.07	**0.03**
BCC-CSM2-MR	0.09	**0.04**	0.22	**0.04**	0.09	**0.04**	0.06	**0.02**
CMCC-ESM2	0.12	**0.03**	0.24	**0.03**	0.28	**0.08**	0.17	**0.05**
CNRM-ESM2-1	0.11	**0.03**	0.09	**0.04**	0.10	**0.05**	0.17	**0.11**
FGOALS-g3	0.15	**0.05**	0.14	**0.03**	0.30	**0.10**	0.14	**0.09**
GFDL-ESM4	0.17	**0.07**	0.27	**0.04**	0.11	**0.05**	0.11	**0.04**
INM-CM5-0	0.22	**0.07**	0.32	**0.07**	0.25	**0.09**	0.20	**0.11**
KACE-1-0-G	0.09	**0.06**	0.20	**0.06**	0.08	**0.03**	0.08	**0.03**
MIROC6	0.09	**0.04**	0.19	**0.06**	0.18	**0.06**	0.19	**0.07**
MPI-ESM1-2-HR	0.15	**0.05**	0.23	**0.05**	0.15	**0.09**	0.07	**0.03**
MPI-ESM1-2-LR	0.18	**0.07**	0.31	**0.08**	0.20	**0.12**	0.12	**0.04**
MRI-ESM2-0	0.18	**0.06**	0.07	**0.03**	0.17	**0.05**	0.15	**0.05**
NorESM2-LM	0.22	**0.11**	0.31	**0.07**	0.15	**0.12**	0.15	**0.09**
average	0.16	**0.07**	0.25	**0.05**	0.17	**0.07**	0.14	**0.06**
